# Creation of a rating scale to teach Less Invasive Surfactant Administration (LISA) in simulation

**DOI:** 10.1186/s12909-024-05118-6

**Published:** 2024-02-14

**Authors:** Hélène Rostoker, Bernard Guillois, Amaya Caradec, François Lecomte, Denis Oriot, Clément Chollat

**Affiliations:** 1 Sorbonne Université, Department of Neonatal Paediatrics, Trousseau Hospital, APHP, F-75012 Paris, France; 2 Normandie Simulation Center in Health Care (NorSimS), 14033 Caen, France; 3 Université Caen Normandie, 14032 Caen, France; 4 P2ULSE, Hôpital Trousseau, APHP centre – Sorbonne Université, Paris, France; 5 Service des urgences de l’hôpital Cochin, APHP centre, Université de Paris Cité, Paris, France; 6 ABS Lab, Simulation Laboratory, Faculty of Medicine of Poitiers, Poitiers, France; 7 Université Paris Cité, Inserm, NeuroDiderot, F-75019 Paris, France

**Keywords:** Neonatology, Respiratory distress syndrome, Rating scale, LISA, Simulation

## Abstract

**Background:**

Simulation-based training is gaining increasing prominence in neonatology training. The Less Invasive Surfactant Administration (LISA) method is starting to be taught in simulation. The aim of this educational study was to develop and validate a rating scale for teaching the LISA method in simulation.

**Methods:**

The Downing framework was used to create this performance-rating scale. A first version of the scale was submitted to 12 French and Belgian experts to obtain their opinions. Consensus was reached using a modified Delphi method. The performance of 40 pediatricians was then evaluated with this scale on a preterm neonate manikin simulating a neonatal respiratory distress syndrome. Each run was evaluated using the scale by two independent observers based on video recordings.

**Results:**

The Cronbach alpha score of the rating scale was 0.72. The intraclass correlation coefficient (ICC) was 0.91 and the scores between raters were not significantly different. Finally, this rating scale correctly distinguished the experienced from the inexperienced learners (p < 0.01).

**Conclusions:**

This rating scale is one of the first rating scales for the evaluation and teaching of the LISA method in simulation. This tool has ample potential for use in clinical practice to evaluate the performance of surfactant administration in preterm neonates.

## Contributions to the literature

- Less invasive surfactant administration (LISA) is now the standard for surfactant administration and must be learned by neonatologists.
- Simulation training is an appropriate way to teach this procedure.
- For this study, we created and validated the first scale for teaching and assessing the LISA procedure in simulation. This high-quality tool is reliable, reproducible, and easy to use

## Background

Respiratory distress syndrome (RDS) particularly impacts preterm neonates and affects nearly all infants born at 28 weeks of gestation or less. The approach to respiratory support in preterm infants with RDS has evolved toward more frequent use of noninvasive methods, resulting in improved clinical outcomes and potentially lower hospitalization costs [1–3]. In keeping with this trend, Less Invasive Surfactant Administration (LISA) techniques have become increasingly common. This technique involves injecting surfactant into the trachea through a thin catheter during laryngoscopy while maintaining noninvasive ventilation over the child's nose. Once the surfactant is injected, the catheter is removed [4, 5]. The main advantage of this method is that it avoids intubation and all the associated risks [6]. The LISA procedure reduces the median days on mechanical ventilation, intubation-related lung injury, and oxygen requirement at 28 days of life compared to the INSURE method (Intubation SURfactant Extubation), which is a less invasive method [7, 8]. The LISA method also reduces the composite score for death and/or bronchopulmonary dysplasia. Since the latest update of the European Consensus Guidelines on the management of RDS in 2019, the LISA technique has been considered to be the recommended method for surfactant administration, provided that clinicians have sufficient experience [9, 10]. Recent reports indicate that only 8% of neonatologists in the United States and 11% of neonatal units in England claim to routinely use this method [11]. This could be partly explained by a lack of consensus regarding clinical practice and training for pediatricians to acquire LISA competence [12]. Training neonatologists in this new technique is essential, and simulation is a particularly appropriate approach to learning it. To validate simulation skills, a criterion-referenced assessment with a rating scale is required. The tools must be consensual and have good reliability and validity. The aim of this study was to develop and validate a rating scale for teaching the LISA method in simulation.

## Methods

### Institutional review board

This project was approved by the Research Ethics Committee of Sorbonne University, Paris, France (CER-2022-028). All participants received an information letter. Informed consent was obtained from all subjects and participants signed a waiver of image rights.

This work used the Downing method [13]. Five steps are necessary for the construction and validation of an assessment tool:

### Development of the rating scale

**Fig. 1:**
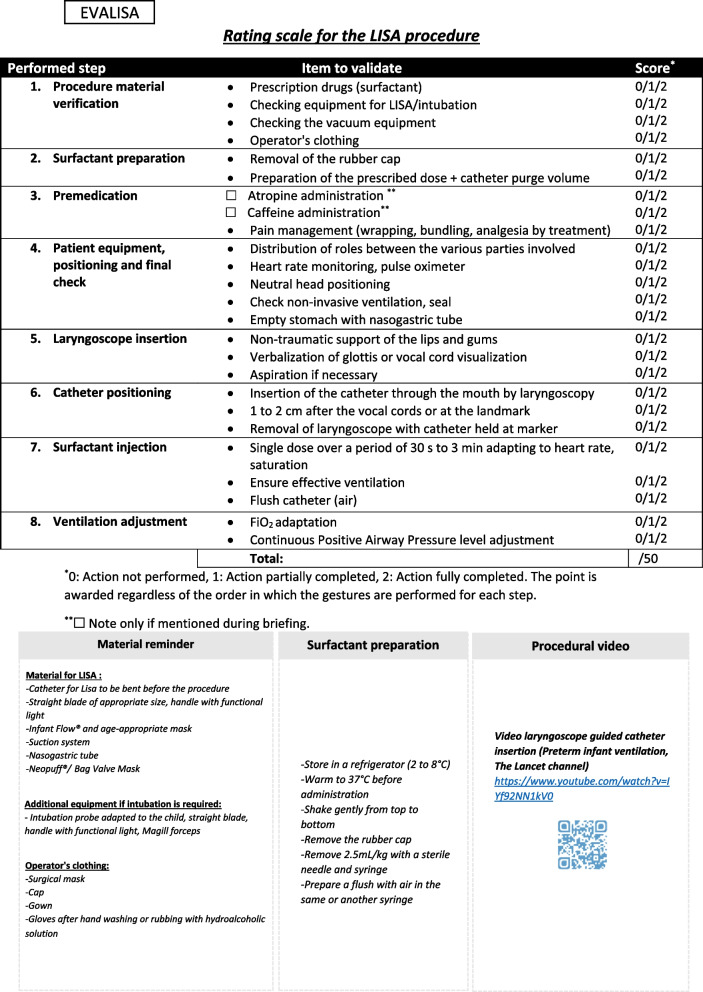
Rating scale for the LISA procedure

-The content of the rating scale must be based on good clinical experience and a review of the literature. Each item of the rating scale must be related to the subject studied by the tool. The first version of the rating scale was drafted by HR and CC. The rating scale was then sent to several experts in the method under study. To constitute the LISA expert panel in charge of assessing the relevance of each item of the scale, 12 neonatologists were recruited in France and Belgium who had a high level of expertise (more than 5 years of practice) in regard to the LISA method and in simulation teaching. They were recruited by mail. They worked in accordance with the modified Delphi method. This method is used to reach a consensus between multiple experts regarding a single question [14]. Following this modified Delphi method, the experts rated each item from 0 to 6. The responses of each expert were anonymized using a letter of the alphabet. In keeping with the modified Delphi method, each item with a median score of less than 4 was modified, and this step was repeated until all the items had been validated by the experts. After a first round of assessment by the experts, all items on the rating scale had medians above 4. Nevertheless, the wording of some of the items was modified according to the experts' comments, and the rating scale was submitted to them a second time. Figure 1 shows the rating scale developed with the expert group. The final scale is composed of 25 items divided into eight categories.

The different categories represent the main stages of surfactant administration using the LISA method. These stages specify the performance of the procedure itself and all the elements of preparation prior to the performance of the procedure.

Each item is scored 0, 1, or 2 depending on whether the participant failed, passed part of the item, or passed the entire item perfectly. The point is awarded regardless of the order in which the gestures are performed for each step.

The total score is out of 50 and can easily be reduced to 100.

Below the rating scale, a reminder of the necessary equipment as well as the doses of medication and a link showing the insertion of the catheter between the vocal cords was added to facilitate the use of the scale in the departments after the training.

The drugs required for sedation were not specified to allow each center to define their own sedation protocol.

-The response process: The first version of the rating scale was used in the simulation so that the observers knew how to rate the simulator runs. We carried out a first simulation with a physician who had already been trained in the LISA method within P2ULSE (Plateforme Pluridisciplinaire Hospitalo-Universitaire de e-Learning et de Simulation de l'Est parisien). No items were changed following this test simulation session. Some items appeared to be difficult to evaluate, but they were kept intact in the scale for training reasons. This point will be addressed in the discussion section. The first observer used the scale to assess this run and then trained the second observer.

### Validation process for the obtained rating scale

The last three points of the Downing method require simulation sessions.

#### Population

The aim was to recruit forty participants. The number of participants was based on previously published studies [15, 16]. The simulations were offered on a voluntary basis as training for LISA. All participants were pediatric residents or senior physicians from Paris and its surrounding areas. They were recruited by mail or phone. Those classified as “inexperienced” were those who had performed the entire LISA procedure less than five times. Those classified as “experienced” were participants who had performed the LISA procedure at least five times.

#### Simulation session material

All the simulation sessions took place at the Trousseau Hospital (Assistance Publique des Hôpitaux de Paris (APHP), Paris), in the P2ULSE (Plateforme Pluridisciplinaire Hospitalo-Universitaire de e-Learning et de Simulation de l'Est parisien) simulation laboratory in February 2022. A "Premature Ann" manikin (Laerdal®, Stavanger, Norway) was used for the simulation sessions. It is a 25-weeks-of-gestation low-fidelity manikin, including an exact replica of the airways and body size [17]. The design provides a degree of realism to procedures such as LISA. The environment of the simulation platform was that of a Level II maternity ward [18], with a heated radiant table, a T-piece resuscitator (Neopuff®), surfactant with preparation materials (syringe, needles), a fine catheter (LISACath®, Chiesi), intubation materials, and a laryngoscope with a Magill 00 blade. The sessions were filmed using three video cameras and recorded on a computer and a secure hard drive.

#### The simulation step

As usual, the simulation sessions took place in four steps: prebriefing, briefing, simulation, and debriefing.

The pre-briefing consisted of a PowerPoint® presentation explaining the relevance of LISA and all the steps required for the procedure. The participants were then given a presentation of the simulation room, in particular the manikin and the equipment available.

The briefing consisted of a succinct presentation of the medical situation. Each participant was asked to endorse the role of a physician belonging to the French pediatric emergency medical service transport team, arriving at a Level II maternity hospital where a 600 g newborn infant had just been born. The patient was in respiratory distress on oxygen and continuous positive airway pressure of 5 cm H_2_O. A facilitator was present in the simulation room and played the role of an emergency transport nurse. The participant was then invited to enter the simulation room. The debriefing was not carried out as a group after each run, as the other participants had to remain unaware of the scenario used. Each participant was invited to debrief individually with the two instructors.

Videos of the simulation sessions were then independently viewed and evaluated by the two blinded raters (HR and BG). The evaluators used the rating scale to rate each participant's performance.

All this was to allow the three next steps of the Downing method to be performed:

- Internal structure study: The aim of this step was to study the reliability of the rating scale. To do this, we need to evaluate the scores that the two observers gave to the participants during the simulation sessions. In this section, we assessed whether the number of items in the scale was correct and to what extent items in the same group were similar to each other.
- Comparison: The aim of this step was also to evaluate the reliability of the scale under modification of its score according to different groups of participants. Students partake in simulation sessions, and two raters score their performance using the rating scale. Two different observers should be able to assign a similar score to the same simulator run.
- Consequence: An evaluation tool must be able to distinguish between failures and successes, in other words between the degree to which participants are familiar with this technique. This last step reinforces the validity of the rating scale.

### Statistical analysis

Reliability analysis included internal consistency testing using a Cronbach’s alpha (CA) test and interrater reliability analysis using intraclass correlation coefficient (ICC), linear regression and its coefficient. F-test or t-test were used to compare scores, as appropriate. Validity analysis included a comparison of the mean scores obtained by technical novices and experts using a t-test.Statistical significance was assumed below a *p*-value threshold of 0.05. All the statistical tests were carried out on Excel® version 2205, published by Microsoft®.

## Results

### Description of the population

Forty participants were recruited to attend the simulation sessions. They were all resident or senior pediatricians working in Paris or the surrounding areas. They all volunteered to participate in the simulation sessions, which took place over 7 sessions in February 2022. There were 5 to 6 participants per simulation session.

The average age of the participants was 31.6 years, and there were 11 men and 29 women. Their characteristics are presented in Table 1. Six trainees were "experienced" in the LISA technique and 34 were "inexperienced" according to our definition.

**Table 1:** Characteristics of the population

**Data from participants in the simulation sessions**	***N* = 40** **n (%)**
**Residents**	**21 (52.5%)**
1^st^ year	1 (5.0%)
2 ^nd^ year	2 (5.0%)
3 ^rd^ year	4 (10%)
4 ^th^ year	13 (32.5%)
**Senior physicians**	**19 (47.5%)**
Neonatologists	13 (32.5%)
Pediatricians from PICU	4 (10%)
Pediatricians (other specialties)	2 (5%)
**LISA experience**	
0 times	24 (60%)
< 5 times	10 (25%)
5 to 10 times	2 (5%)
> 10 times	4 (10%)
**Intubation experience**	
< 5 times	13 (32.5%)
5 to 10 times	10 (25%)
> 10 times	17 (42.5%)

### Statistical validation of the rating scale

#### Reliability analysis

The overall CA was 0.72 for the entire rating scale. A CA coefficient above 0.9 indicates that the rating scale has repetitive items. A score below 0.7 indicates poor internal consistency and could be explained by discrepancies between items or a lack of items in the analysis.

**Fig. 2:**
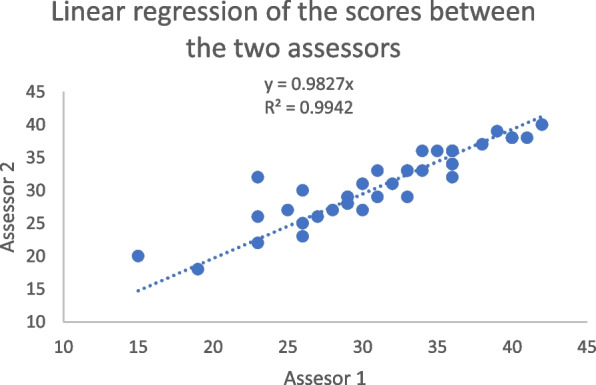
Linear regression of the scores between the two assessors

The overall ICC for the scale was 0.91. There was no significant difference between the mean scores of the two raters (31.5, standard deviation = 6.2 versus 31.1, standard deviation = 5.4, *p* = 0.80). In linear logistic regression, the coefficient of determination (R2) of the scores between the two raters was 0.99 (Fig. 2).

#### Validity analysis

The mean score on the rating scale for the LISA procedure was 36.8 for the experienced trainees versus 30.5 for the nonexperienced learners (*p* < 0.001). The average expert score was 36.8/50, or 73.6%. Given the small number of learners defined as “experienced”, the results obtained by the participants according to their intubation experience were compared. Intubation experience was defined as having performed more than 10 intubations. LISA requires the use of a laryngoscope and visualization of the vocal cords. These two parts of the procedure are the most delicate to perform, yet they are essential for intubation. The hypothesis was that participants with experience with intubation would do better on the LISA than those without such experience. The scores were significantly higher for the learners with intubation experience than for those without (33.9 versus 29.7, respectively; *p* = 0.016).

## Discussion

The present study developed a rating scale for the LISA procedure in preterm neonates in simulation by the modified Delphi method. The validation process of the scale (including 40 participants in seven simulation sessions) found good internal consistency (CA=0.72) and very good reliability interraters (R2 0.99 and ICC=0.91).

In pediatrics, several rating scales have already been developed and published. They concern technical skills such as placing an intraosseous catheter [16], carrying out a lumbar puncture [19], or performing intubation in a newborn [20], as well as nontechnical skills such as early identification of sepsis [21] or the announcement of bad news [22]. To our knowledge, this scale is the first developed for teaching the LISA method in simulation. Not all the studies systematically assessed validation as we did. The results are consistent with those obtained in studies that have published recognized tools, such as the study by Oriot et al, with an ICC score of 0.947, or the study by Diaz et al, where the CA score was 0.87. Nevertheless, this tool has some limitations. First, some items of the rating scale could not be properly evaluated ("neutral head position" and "nontraumatic supports"). The first item, "neutral head position," could not be assessed because the low-fidelity manikin used had the head in a neutral position from the start. The participants were informed during the prebriefing that it was necessary to verbally state the procedures that could not be performed in the simulation. The observers were informed that part of the evaluation would be based on the oral instructions given by the participants. Many participants did not verbally report that they were checking the child's head position. The assessors could, therefore, not evaluate whether the neutral position of the manikin’s head was the participant's choice during the simulation session, which was related to the specific technical characteristics of the manikin. The second item that could not be assessed was the item "nontraumatic laryngoscope insertion". This difficulty with evaluation was due to the angle of the cameras with which the video of the sequence was recorded. In addition, the traumatic nature of laryngoscope insertion could be subjective, as there may be no visible lesion in the manikin's mouth.

If this scale is to be used for training, it will be necessary to place a camera close to the mouth of the manikin to check the operator's support. These two items have been retained in the rating scale because they are essential elements in the performance of surfactant injection.

Indeed, this rating scale was created with a double objective. It had to be a criterion-referenced assessment scale used for learners and, therefore, had to be valid and reliable to allow objective evaluation of the learners' skills. It was also intended to be a teaching aid for beginners.

More frequent use of simulation for training in the healthcare field has been encouraged by the publication of the report "To Err is Human" [23]. The aim of systematically using simulation in healthcare training is twofold: to reduce the risk of error and to avoid performing the procedure on a patient for the first time. However, performing the procedure on patients remains the final objective after simulation training. Some elements, such as "neutral head position", are difficult to assess in a criterion-referenced manner in simulation but must nonetheless be taught to the student so that they can perform the procedure correctly on patients after having learned it in simulation.

The LISA method of surfactant delivery has become the standard of care in neonatology. This procedure is highly technical and requires appropriate training, which is best provided by manikin simulation. As part of this training, it is necessary to develop a rating scale for this procedure to assess learners in a criterion-referenced manner.

In the present scale, there are eight steps, with a total of 25 items. Each item is scored either 0, 1, or 2. It was decided to use a 0, 1, or 2 score because some items contain several assessable elements, such as dressing or pain management.

During the validation process of this scale, it was found that there was a significant difference between those already trained in LISA versus those naive to the technique. The mean result of the participants already trained in LISA was 36.8/50, i.e., a success rate of 73.6% for the items. This could suggest that a success rate at least of 73.6% appears to be predictive of satisfactory achievement of the LISA method.

Moreover, this rating scale also contains a "reminder" part comprising the materials needed to perform the procedure as well as the medication. This latter part, added to the checklist of the scale per se, could be considered to be a cognitive aid that makes the LISA assessment tool (rating scale + reminder) usable in neonatal intensive care units. This possibility underlines the proximity and educational continuity between the simulation platform and real-life practice.

The present work with the experts revealed that some elements such as sedation and the use of atropine before the procedure are still a matter of debate. Sedation before the procedure is essential; however, studies are still needed to define the most appropriate sedation for the procedure and the type of patient. Randomized controlled trials are underway, and the French Society of Neonatology has recently recommended the use of propofol for LISA sedation [24, 25]. In the literature, there are no randomized trials evaluating the administration of atropine before LISA. In observational studies, no severe adverse events have been reported [24, 26]. The French Society of Neonatology suggests that atropine should be administered preventively or in the event of bradycardia [25]. It was decided to make these two items (sedation and atropine use) optional for use according to local practice. If these items are to be assessed, they should be presented during the prebriefing.

In a follow-up study, it would be of interest to apply this technique in an intensive care unit and to use the evaluation rate to perform the training. Similarly, to assess the pedagogical contribution of this scale, it might be interesting to evaluate the success rate after the accelerated training of novices in the simulation technique. The novices could then perform the procedure once they obtained the average score obtained by the experts in simulation, i.e., at least 36.8/50 (or 73.6%).

## Conclusions

The LISA method is now the recommended first-line method of surfactant administration. It is not yet used in all neonatal units, probably in part because it is not routinely taught. This rating scale, presented here, is the first scale to evaluate and teach the LISA method in simulation. The psychometric testing of the scale yielded good reliability and validity. This rating scale could be used to train beginners in the LISA method. A score of more than 36/50, i.e., a 72% success rate, appears to indicate a good ability to perform the procedure. Training future physicians dealing with preterm infants to perform LISA in simulation is crucial to improve the quality of care. Following this study, research is currently underway to define a minimum passing score for this criterion referenced evaluation instrument, with a view to its use in summative evaluation.

## Data Availability

The datasets used and analyzed during the current study are available from the corresponding author on reasonable request.

## Abbreviations

APHP: Assistance Publique des Hôpitaux de Paris
CA: Cronbach’s alpha
ICC: Intraclass Correlation Coefficient
LISA: Less Invasive Surfactant Administration
P2ULSE: Plateforme Pluridisciplinaire Hospitalo-Universitaire de e-Learning et de Simulation de l'Est Parisien
RDS: Respiratory Distress Syndrome
R2: Coefficient of determination
